# Effect of Sowing Methods and NPK Levels on Growth and Yield of Rainfed Maize (*Zea mays* L.)

**DOI:** 10.1155/2015/198575

**Published:** 2015-05-18

**Authors:** Shamim Gul, M. H. Khan, B. A. Khanday, Sabeena Nabi

**Affiliations:** Division of Agronomy, Sher-e-Kashmir University of Agricultural Sciences and Technology of Kashmir, Shalimar, Srinagar 191 121, India

## Abstract

To investigate the response of rainfed maize to sowing methods and NPK levels, an experiment was undertaken during kharif of 2011 and 2012 at Dryland (Kerawa) Agriculture Research Station, Sher-e-Kashmir University of Agricultural Sciences and Technology of Kashmir, Budgam. The experiment was laid out in a randomized block design with combination of 2 sowing methods (flat sowing, 75 cm apart rows, and ridge sowing, 75 cm apart ridges) and 3 fertility levels (60 : 40 : 20, 75 : 50 : 30, and 90 : 60 : 40 N : P_2_O_5_ : K_2_O kg ha^−1^) with three replications. Various growth characters, namely, plant height, leaf area index, dry matter accumulation, number of days to different phenological stages, and yield, and yield contributing characters namely, cob length, number of grains cob^−1^, cob diameter (cm), and 100-seed weight (g), were significantly higher with S_2_ over S_1_ during both the years of experimentation. Fertilizer levels F_3_ (90 : 60 : 40) and F_2_ (75 : 50 : 30) at par with one another produced significant increase in growth and yield characters, namely, plant height, leaf area index, dry matter production at different growth stages, cob length, number of cobs plant^−1^, number of grains cob^−1^, and 100-seed weight over F_1_ (60 : 40 : 20). Significantly higher grain yield was recorded with fertilizer level F_3_ (90 : 60 : 40) being at par with F_2_ (75 : 50 : 30) and showed significant increase over F_1_ (60 : 40 : 20) with superiority of 5.4 and 5.7 per cent during 2011 and 2012, respectively. The findings of the study concluded that ridge method of sowing of maize with NPK levels of 75 : 50 : 30 kg ha^−1^ showed better performance of crop in terms of growth, yield, and yield attributes.

## 1. Introduction

Among the modern agromanagement practices sowing method and fertilizer application are imperative for boosting the growth and production of maize especially under rainfed conditions. Considerable work has been reported on these aspects but efforts are still required to improve these techniques for getting maximum yield. Planting technique has a great role to play in increasing maize yield. Our farmers generally use the old broadcast method of sowing that has so many disadvantages, that is, uneven distributions of seeds, depth, and seed lying scattered being picked up by birds. Improved planting method may lead to increased production of maize which will result in attaining self-sufficiency in food and feed [1]. Planting technique not only ensures proper adjustment and optimum plant population in the field but also enables the plants to utilize the land and other input resources more efficiently and resolutely towards growth and development.

Nitrogen (N) is a vital plant nutrient and a major determining factor required for maize production [2]. It is very essential for plant growth and makes up 1–4% of dry matter of the plants. Nitrogen is a component of protein and nucleic acids and when N is suboptimal, growth is reduced [3]. Its availability in sufficient quantity throughout the growing season is essential for optimum maize growth. It is also a characteristic constituent element of proteins and also an integral component of many other compounds essential for plant growth processes including chlorophyll and many enzymes. It also mediates the utilization of phosphorus, potassium, and other elements in plants [4]. Optimal amount of these elements in the soil cannot be utilized efficiently if nitrogen is deficient in plants. Therefore, nitrogen deficiency or excess can result in reducing maize yields. Phosphorus is another essential nutrient required to increase maize yield [5]. Consequently, the lack of phosphorus is as important as the lack of nitrogen limiting maize performance. Phosphorus plays an important part in many physiological processes that occur within a developing and maturing plant. It is involved in enzymatic reactions in the plant. Phosphorus is an essential factor for cell division because it is a constituent element of nucleoproteins which are involved in the cell reproduction processes. It is also a component of a chemical essential to the reactions of carbohydrate synthesis and degradation [6]. It is important for seed and fruit formation and crop maturation. Phosphorus hastens the ripening of fruits thus counteracting the effect of excess nitrogen application to the soil [7]. It helps to strengthen the skeletal structure of the plant thereby preventing lodging. It also affects the quality of the grains and it may increase the plant resistance to diseases. However, the requirement and utilization of these nutrients (nitrogen and phosphorus) in maize depend on environmental factors like rainfall, varieties, and expected yield.

Potassium is an essential nutrient and is also the most abundant cation in plants. It plays essential roles in enzyme activation, protein synthesis, photosynthesis, osmoregulation, stomatal movement, energy transfer, phloem transport, cation-anion balance, and stress resistance. A major limitation for plant growth and crop production under rainfed condition is soil water availability. Plants that are continuously exposed to drought stress can form reactive oxygen species (ROS), which leads to leaf damage and, ultimately, decreases crop yield. During drought stress, root growth and the rates of K^+^ diffusion in the soil towards the roots are both restricted, thus limiting K acquisition. The resulting lower K concentrations can further depress the plant resistance to drought stress, as well as K absorption. Maintaining adequate plant K is, therefore, critical for plant drought resistance. A close relationship between K nutritional status and plant drought resistance has been demonstrated [6]. Keeping in view the above review, the present study was undertaken to determine the effect of sowing methods fertility levels on growth, yield, and yield attributes of rainfed maize.

## 2. Materials and Methods

The investigation was conducted during kharif of 2011 and 2012 at Dryland (*Kerawa*) Agriculture Research Station, Sher-e-Kashmir University of Agricultural Sciences and Technology of Kashmir, Budgam. The area lies between 34° 0.8′ N latitude and 74° 83′ E longitude at an altitude of 1587 meters above the mean sea level. The mean maximum temperature ranged from 24.3 to 31.5°C and the minimum from 9.7 to 17.60°C during the first growing season and from 21.2 to 32.2°C and from 8.2 to 19.8°C during the second growing season. The total rainfall received during the entire growing seasons of 2010-11 and 2011-12 amounted to 383.70 mm and 426.10 mm, respectively. The experiment was laid out in a randomized block design with combination of 2 sowing methods (flat sowing, 75 cm apart rows, and ridge sowing, 75 cm apart ridges) and 3 fertility levels (60 : 40 : 20, 75 : 50 : 30, and 90 : 60 : 40 N : P_2_O_5_ : K_2_O kg ha^−1^) with three replications. Prior to sowing, the field site was ploughed three times approximately 30 cm deep using a cultivator to destroy offtypes. Fine seed beds were prepared prior to sowing. The maize variety “C-6” was sown at a spacing of 75 cm × 20 cm between rows and plants. The trial was irrigated when required. Full dose of phosphorus and potassium and 1/3rd of nitrogen were band placed as per the treatment just before sowing of seed. The remaining nitrogen was top dressed in two equal splits at knee high and tasselling stages. Fertilizer nitrogen, phosphorus, and potassium were applied through urea, diammonium phosphate, and muriate of potash, respectively.

Growth parameters, namely, plant height (cm), leaf area index, days to reach different phenological stages (knee high, tasseling, silking, and maturity), and dry matter production (q ha^−1^), were recorded from penultimate rows of each plot. The leaf area of each leaf was calculated by multiplying the length and maximum width. The value thus obtained was multiplied by a constant 0.73309 to get actual leaf area and then leaf area index (LAI) was calculated by dividing the leaf area per plant by ground surface provided to each plant (1200 sq. cm). Five randomly selected plants in each plot were tagged for various periodic observations. The same procedure was followed for both the years. For recording of data on yield attributes, namely, cob length (cm), cobs plant^−1^, grains cob^−1^, cob diameter (cm), number of rows cob^−1^, and 100-grain weight (g), the number of cobs of five randomly selected plants from each plot was used. The grain yield of each net plot was thoroughly cleaned and sun-dried. The yield from each plot was recorded separately as kg plot^−1^ and then converted in q ha^−1^. After removal of the cobs from stalks in each net plot, the stalks were weighed to determine the stover yield in q ha^−1^.

The data obtained in respect of various observations were statistically analyzed by the method described by Cochran and Cox [8]. The significance of “F” and “T” was tested at 5% significance level. The critical difference was determined when “F” test was significant.

## 3. Results and Discussion

### 3.1. Fertility Levels

#### 3.1.1. Growth Parameters

Data (Table 1) indicates that the plant height at different stages of growth showed significant increase with fertility level up to F_2_ (75 : 50 : 30) compared to F_1_ (60 : 40 : 20); however, the fertility level F_3_ (90 : 60 : 40) did not differ from F_2_ (75 : 50 : 30). This could be attributed to a mere fact that higher rates of nitrogen may have caused rapid cell division and elongation. Sarwargaonkar et al. [10] reported significant increase in the plant height of kharif maize with 100% recommended fertilizer dose (RFD) compared to 75% RFD. Leaf area index, a vital photosynthetic character, was found significantly affected by fertility levels. Increase in level of fertility from F_1_ (60 : 40 : 20) to F_2_ (75 : 50 : 30) significantly improved leaf area index at different crop growth stages and beyond F_2_ (75 : 50 : 30) level, the difference was nonsignificant. Maximum leaf area was recorded at tasseling stage. F_2_ (75 : 50 : 30) level might have provided sufficient nitrogen to the crop for rapid cell division and cell elongation thereby resulting in increased leaf area. Shivay and Singh [9] also found improvement in leaf area index with increasing levels of nitrogen. The decrease in leaf area index of crop irrespective of fertility levels after tasseling could be attributed to senescence of lower leaves. It was found that F_3_ (90 : 60 : 40) significantly increased number of days for crop to reach different phenological stages. Increased dose of nitrogen might have lengthened the vegetative phase of the crop, thereby delaying the reproductive period of the crop. The study revealed a gradual increase in dry matter production of crop from knee high to maturity stage irrespective of fertility levels and the magnitude of increase was highly pronounced from knee high to tasseling stage. This could be attributed to vigorous growth of crop in terms of gain in plant height and higher number of functional leaves per plant. Moreover, higher leaf area index of the crop at tasseling indicates higher crop growth rate from knee high to tasseling stage thereby resulting in higher dry matter production which could have resulted from higher plant height and higher number of functional leaves. Fertility level F_2_ (75 : 50 : 30) significantly improved dry matter production over F_1_ (60 : 40 : 20) but was nonsignificant with F_3_ (90 : 60 : 40) level. Higher leaves and higher LAI paved the way for more production of photosynthetic dry matter. Similar results have also been reported by Sarwargaonkar et al. [10].

#### 3.1.2. Yield and Yield Attributes

The investigation revealed that yield contributing characters, namely, cob length and diameter, number of cobs per plant, grain rows, number of grains per cob, and 100-grain weight, increased significantly up to F_2_ (75 : 50 : 30) level beyond which difference was nonsignificant (Table 2). Higher cob length and diameter obtained at F_2_ (75 : 50 : 30) level might be due to sufficient supply of nitrogen to the crop because nitrogen being an essential constituent of plant tissue is involved in cell division and cell elongation. Moreover, higher leaf area index values noticed at F_2_ (75 : 50 : 30) level mean the production of more photosynthates leading to increase in grain number and weight of grains. Rasheed et al. [11] have also reported similar findings. Besides, increase in 100-grain weight might be due to enhancement in source efficiency as well as sink capacity [12].

The study revealed that treatments influenced the seed yield significantly during both years of experimentation (Table 3). Fertilizer level F_3_ (90 : 60 : 40) being at par with F_2_ (75 : 50 : 30) recorded the increased yield over F_1_ (60 : 40 : 20) with superiority of 5.4 and 5.7 per cent over F_1_ during 2011 and 2012, respectively. The yield components, namely, cobs per plant, grains per cob, and grain weight, increased significantly up to F_2_ (75 : 50 : 30) level; thereby the combined effect of these components resulted in yield increase. A similar effect of fertilizer levels on maize yield and its components was reported by Bakht et al. [13] and Maqsood et al. [12]. The stover yield also showed increasing trends as that of grain yield. Fertilizer levels F_3_ (90 : 60 : 40) and F_2_ (75 : 50 : 30) at par with one another recorded significant increase in stover yield over F_1_ (60 : 40 : 20) during both the years of the study. The higher uptake of nutrients by the crop produced higher LAI meaning more production of photosynthates leading to higher dry matter production in terms of grain and stover yield. Abdullah [14] and Ghaffar et al. [15] also reported similar findings.

### 3.2. Sowing Methods

#### 3.2.1. Growth Parameters

The study revealed significant enhancement in plant height with S_2_ treatment (Table 1). In fact, S_2_ (ridges) provides loose fertile soil with more aeration and moisture availability; therefore, improved soil condition along with better uptake of nutrients might have provided better environment to crop resulting in improved plant height. Khan et al. [16] have also reported maximum plant height under ridge sowing. It was also found that ridge sowing resulted in higher leaf area index of crop at different stages. Better and developed root system in loose fertile soil of ridges might have improved water availability and nutrient uptake resulting in maximum leaf area index. Earlier Amin et al. [1] have also reported higher leaf area index of maize under ridge sowing due to enhanced water and nutrient availability. The ridges significantly increased the period for crop to reach different phenological stages. This could be attributed to better uptake of nutrients especially nitrogen in loose fertile soil of ridges because nitrogen is known to lengthen vegetative period of crop thereby delaying maturity. The study also revealed significant increase in dry matter production. Large assimilatory system produced by higher leaf area index due to higher photosystem together with higher plant height and higher number of functional leaves under ridge system might have increased dry matter production. These results are in line with those of Hussain et al. [17] and Khan et al. [16].

#### 3.2.2. Yield and Yield Attributes

The study revealed that ridge sowing significantly improved cob length and diameter, grain rows, number of grains per cob, and 100-grain weight (Table 2). Higher cob length and diameter might be attributed to higher leaf area index and plant height in fertile loose soil of ridges. Further, more water and nutrient availability resulting in high leaf area index providing more availability of assimilates might have improved grain rows and number of grain rows per cob and 100-seed weight. Rasheed et al. [11] and Khan et al. [16] have also obtained similar results.

Grain yield is the product of cobs per unit area, grain per cob, and grain weight. During the study it was found that cobs per plant, grains per cob, and 100-grain weight were maximum in ridge system thereby because their cumulative effect increased the grain yield (Table 3). The promotive effect of ridges plantation on grain yield and its components has also been reported by Rasheed et al. [11] and Khan et al. [16]. The stover yield varied significantly among methods of sowing. Ridge sowing produced significantly more stover yield than flat sowing. This might be due to the favourable soil condition created by ridges resulting in better root development thereby enabling plants to uptake more moisture and nutrients to produce high LAI meaning bigger assimilatory system and hence more dry matter production leading to higher biological yield. These results are in accordance with the findings of Raymond et al. [18].

Various aspects of the present investigation and observation generated showed that all growth and yield and yield attributing traits were discernibly influenced by manipulation in sowing methods and NPK level. Results clearly suggested that, for temperate environment of Kashmir Valley, the application of 75 kg N ha^−1^, 50 kg P ha^−1^, and 30 kg K ha^−1^ under ridge method of sowing was found to be an appropriate treatment for growing rainfed maize and can be recommended for farmers of Kashmir Valley.

## Figures and Tables

**Table 1:** Growth characters of maize at different growth stages as affected by sowing methods and NPK levels.

Treatments	Growth stages (2011)				Growth stages (2012)			
	Knee high	Tasseling	Silking	Maturity	Knee high	Tasseling	Silking	Maturity
Plant height (cm)								
S_1_ (flat sowing)	54.28	159.83	166.36	171.35	55.08	160.60	168.35	172.42
S_2_ (ridge sowing)	56.72	165.38	173.22	177.45	57.41	166.80	173.23	178.24
SE(m) ±	0.65	0.76	0.93	0.93	0.55	0.83	0.90	0.96
CD (*p* = 0.05)	1.89	2.34	2.87	2.89	1.71	2.56	2.78	2.98
F_1_ (60 : 40 : 20)	51.28	157.63	163.94	165.47	52.25	158.17	162.59	167.22
F_2_ (75 : 50 : 30)	57.04	164.28	171.07	177.31	57.25	165.07	173.36	177.92
F_3_ (90 : 60 : 40)	58.33	165.91	174.36	180.42	59.23	167.86	176.42	180.85
SE(m) ±	0.75	0.93	1.15	1.15	0.68	1.02	1.11	1.19
CD (*p* = 0.05)	2.33	2.89	3.54	3.57	2.11	3.16	3.44	3.68

Leaf area index								
S_1_ (flat sowing)	1.58	2.44	2.18	1.66	1.61	2.48	2.22	1.67
S_2_ (ridge sowing)	1.77	2.73	2.54	1.92	1.79	2.79	2.59	1.89
SE(m) ±	0.04	0.03	0.04	0.03	0.03	0.03	0.03	0.03
CD (*p* = 0.05)	0.14	0.10	0.12	0.12	0.09	0.11	0.11	0.09
F_1_ (60 : 40 : 20)	1.12	1.79	1.66	1.04	1.15	1.93	1.60	1.10
F_2_ (75 : 50 : 30)	1.93	2.96	2.70	2.16	1.96	2.96	2.78	2.06
F_3_ (90 : 60 : 40)	1.96	3.01	2.72	2.17	1.98	3.00	2.82	2.19
SE(m) ±	0.05	0.04	0.04	0.04	0.03	0.04	0.04	0.03
CD (*p* = 0.05)	0.17	0.12	0.15	0.14	0.11	0.13	0.13	0.11

Dry matter production (q ha^−1^)								
S_1_ (flat sowing)	6.83	41.21	66.86	98.72	7.00	43.52	69.13	103.47
S_2_ (ridge sowing)	7.10	46.27	72.01	109.09	7.45	47.15	74.46	110.77
SE(m) ±	0.04	0.66	0.87	1.14	0.12	0.69	0.93	1.22
CD (*p* = 0.05)	0.13	2.05	2.68	3.52	0.39	2.14	2.86	3.76
F_1_ (60 : 40 : 20)	6.33	40.98	65.71	98.87	6.31	41.92	65.93	99.72
F_2_ (75 : 50 : 30)	7.23	44.91	70.69	106.19	7.46	45.99	73.11	108.97
F_3_ (90 : 60 : 40)	7.34	45.32	71.90	106.63	7.91	48.11	76.35	112.68
SE(m) ±	0.05	0.82	1.07	1.41	0.15	0.85	1.14	1.50
CD (*p* = 0.05)	0.16	2.53	3.31	4.34	0.48	2.64	3.53	4.64

Days taken to reach different phenological stages								
S_1_ (flat sowing)	37.44	71.49	77.39	122.17	37.94	70.61	78.23	121.06
S_2_ (ridge sowing)	41.86	74.50	80.40	126.25	40.91	74.54	80.68	125.65
SE(m) ±	0.45	0.42	0.39	0.45	0.39	0.45	0.52	0.75
CD (*p* = 0.05)	1.28	1.20	1.11	1.26	1.11	1.26	1.48	2.14
F_1_ (60 : 40 : 20)	36.68	69.25	75.38	120.46	37.42	68.83	76.86	120.61
F_2_ (75 : 50 : 30)	40.18	73.69	79.93	124.71	38.88	73.57	79.45	123.36
F_3_ (90 : 60 : 40)	42.09	76.05	81.26	127.46	41.97	75.32	82.06	126.11
SE(m) ±	0.48	0.45	0.34	0.48	0.48	0.47	0.64	0.94
CD (*p* = 0.05)	1.37	1.29	1.19	1.35	1.37	1.35	1.83	2.64

SE(m): standard error of mean, CD: critical difference.

**Table 2:** Yield contributing characters of maize as affected by sowing methods and NPK levels.

Treatments	Yield attributes					
	Cob length (cm)	Cobs plant^−1^	Grains cob^−1^	Cob diameter (cm)	Number of rows cob^−1^	100-grain weight (g)
2011						
S_1_ (flat sowing)	12.05	1.10	322.91	1.83	15.71	19.67
S_2_ (ridge sowing)	14.40	1.10	334.97	2.15	16.09	20.45
SE(m) ±	0.07	0.01	1.48	0.07	0.17	0.22
CD (*p* = 0.05)	0.22	NS	4.60	0.22	NS	0.69
F_1_ (60 : 40 : 20)	11.49	1.06	318.94	1.73	14.29	19.81
F_2_ (75 : 50 : 30)	13.96	1.12	332.94	2.09	16.31	20.66
F_3_ (90 : 60 : 40)	14.23	1.12	334.94	2.14	17.10	20.71
SE(m) ±	0.10	0.01	1.84	0.09	0.25	0.27
CD (*p* = 0.05)	0.31	NS	5.67	0.28	0.75	0.85

2012						
S_1_ (flat sowing)	13.97	1.10	332.00	1.99	16.13	20.30
S_2_ (ridge sowing)	16.93	1.13	340.00	2.24	16.25	21.87
SE(m) ±	0.22	0.01	1.36	0.03	0.15	0.25
CD (*p* = 0.05)	0.68	0.02	4.20	0.11	NS	0.78
F_1_ (60 : 40 : 20)	14.49	1.08	330.40	1.84	14.88	19.83
F_2_ (75 : 50 : 30)	15.85	1.13	336.80	2.17	16.48	21.58
F_3_ (90 : 60 : 40)	16.19	1.14	340.00	2.25	17.22	21.83
SE(m) ±	0.27	0.01	1.68	0.04	0.23	0.31
CD (*p* = 0.05)	0.84	0.02	5.18	0.13	0.67	0.97

SE(m): standard error of mean, CD: critical difference.

**Table 3:** Seed and stover yield (q ha^−1^) of maize as affected by sowing methods and NPK levels.

Treatments	Seed yield		Stover yield	
	2011	2012	2011	2012
Sowing methods				
S_1_ (flat sowing)	45.67	46.69	68.81	69.95
S_2_ (ridge sowing)	47.45	47.94	70.27	70.81
SE(m) ±	0.15	0.13	0.59	0.48
CD (*p* = 0.05)	0.49	0.43	1.42	0.69

Fertility levels (N : P : K kg ha^−1^)				
F_1_ (60 : 40 : 20)	44.60	45.68	67.75	67.78
F_2_ (75 : 50 : 30)	46.58	47.99	68.59	69.07
F_3_ (90 : 60 : 40)	46.99	48.28	70.27	70.79
SE(m) ±	0.19	0.17	0.73	0.59
CD (*p* = 0.05)	0.59	0.53	1.85	1.04

SE(m): standard error of mean, CD: critical difference.
